# Embryo aggregation regulates *in vitro* stress conditions to promote developmental competence in pigs

**DOI:** 10.7717/peerj.8143

**Published:** 2019-12-13

**Authors:** Pil-Soo Jeong, Seung-Bin Yoon, Mun-Hyeong Lee, Hee-Chang Son, Hwal-Yong Lee, Sanghoon Lee, Bon-Sang Koo, Kang-Jin Jeong, Jong-Hee Lee, Yeung Bae Jin, Bong-Seok Song, Ji-Su Kim, Sun-Uk Kim, Deog-Bon Koo, Bo-Woong Sim

**Affiliations:** 1 Futuristic Animal Resource & Research Center, Korea Research Institute of Bioscience and Biotechnology, Cheongju, Republic of Korea; 2 National Primate Research Center, Korea Research Institute of Bioscience and Biotechnology, Cheongju, Republic of Korea; 3 Department of Biotechnology, College of Engineering, Daegu University, Gyeongsan, Republic of Korea; 4 Primate Resource Center, Korea Research Institute of Bioscience and Biotechnology, Jeongeup, Republic of Korea; 5 Department of Functional Genomics, University of Science and Technology, Daejeon, Republic of Korea

**Keywords:** Pig embryo aggregation, Blastocyst quality, Mitochondrial function, Reactive oxygen species, Endoplasmic reticulum stress

## Abstract

Embryo aggregation is a useful method to produce blastocysts with high developmental competence to generate more offspring in various mammals, but the underlying mechanism(s) regarding the beneficial effects are largely unknown. In this study, we investigated the effects of embryo aggregation using 4-cell stage embryos in *in vitro* developmental competence and the relationship of stress conditions in porcine early embryogenesis. We conducted aggregation using the well of the well system and confirmed that aggregation using two or three embryos was useful for obtaining blastocysts. Aggregated embryos significantly improved developmental competence, including blastocyst formation rate, blastomere number, ICM/TE ratio, and cellular survival rate, compared to non-aggregated embryos. Investigation into the relationship between embryo aggregation and stress conditions revealed that mitochondrial function increased, and oxidative and endoplasmic reticulum (ER)-stress decreased compared to 1X (non-aggregated embryos) blastocysts. In addition, 3X (three-embryo aggregated) blastocysts increased the expression of pluripotency, anti-apoptosis, and implantation related genes, and decreased expression of pro-apoptosis related genes. Therefore, these findings indicate that embryo aggregation regulates *in vitro* stress conditions to increase developmental competence and contributes to the *in vitro* production of high-quality embryos and the large-scale production of transgenic and chimeric pigs.

## Introduction

Pigs are useful experimental animal models because of their close anatomic, genetic, and physiological similarities with humans (Lunney, 2007). Furthermore, pigs are advantageous in biomedical research as disease models (Giraldo, Ball & Bondioli, 2012; Kragh et al., 2009), xenotransplantation studies (Hemann et al., 2012; Watanabe et al., 2012) and chimeric models (Wu et al., 2017). Therefore, it is important to produce high-quality blastocysts to improve the production efficiency of pig models for biomedical research. However, *in vitro-*produced (IVP) embryos remain inferior in terms of developmental competence, including blastocyst formation rate, blastomere number and survival rate, compared to their *in vivo* counterparts (Koo et al., 2004).

Low developmental competence of IVP embryos is mainly attributed to stressful conditions, such as endoplasmic reticulum (ER), oxidative and metabolic stresses during *in vitro* culture (Ali et al., 2017). The ER is an organelle with vital functions in protein folding and secretion and calcium homeostasis (Shiraishi et al., 2006). The accumulation of unfolded or misfolded proteins causes ER stress, which activates cumulative cellular damage including cellular dysfunction and ultimately leads to cell death (Kaneko & Nomura, 2003). Studies have demonstrated that ER stress induces detrimental effects on blastocyst formation and cellular survival in pigs (Kim et al., 2012; Lin et al., 2016). Reactive oxygen species (ROS) are the byproduct of cellular energy metabolism, and induce cellular damage and apoptosis (Halliwell & Aruoma, 1991). ROS-induced developmental arrest and apoptosis in embryo development results in reduced pre-implantation developmental competence and subsequently delayed post-implantation development in bovine (Bain, Madan & Betts, 2011). To overcome ROS-induced stress, researchers have investigated the use of ROS scavengers, such as vitamin C (Jeong et al., 2006), glutathione (Li et al., 2014), and fetal bovine serum (Mun et al., 2017), to enhance developmental competence by decreasing ROS levels. Mitochondria-mediated metabolic stress can also create stressful conditions. Mitochondria are key regulators of cellular energy and act as storage facilities for calcium ions. They are also associated with eukaryotic cellular differentiation, cell death, and growth (McBride, Neuspiel & Wasiak, 2006). Furthermore, mitochondrial number greatly impacts oocyte maturation, fertilization, and embryo development (Babayev & Seli, 2015; Dumollard, Duchen & Carroll, 2007). For instance, the addition of resveratrol with MG132, a proteasomal inhibitor, in porcine *in vitro* maturation medium replenished and improved mitochondrial function and embryo development by activating the expressions of *sirt1*, the gene associated with mitochondrial number (Sato et al., 2014). Other studies have demonstrated that mitochondrial membrane potential, an indicator of cellular metabolic activity, is an important determinant for fertilization and pre-implantation embryonic development in pigs and mice (Romek et al., 2011; Wakefield, Lane & Mitchell, 2011).

The aggregation of several embryos has been shown to be a useful method to improve pre- and post-implantation embryo development. Embryo aggregation was first used to produce chimeric mice (Nagy et al., 1993) and aggregated embryos showed enhanced blastocyst formation rates and total cell numbers in mice (Boiani et al., 2003; Tang & West, 2000), and other animal species, with many benefits to embryo development being reported in equine (Gambini et al., 2014), feline (Moro et al., 2015) and bovine (Zhou et al., 2008). In the porcine model, aggregation increases the total cell number, ICM/TE ratio, *oct4* gene expression in blastocysts (Lee et al., 2007; Terashita et al., 2011), and the efficiency of establishing embryonic stem cell lines (Saadeldin, Kim & Lee, 2015; Siriboon et al., 2015). Despite previous research into embryo aggregation, its relationship with stress conditions remains unclear.

In this study, we hypothesized that embryo aggregation could improve developmental competence by reducing stress conditions during porcine early embryogenesis. To address this, we demonstrated that porcine embryo aggregation using 4-cell stage embryos significantly enhanced developmental competence, including blastocyst formation rate, total cell number, ICM/TE ratio, cellular survival rate, and gene expression. Importantly, our findings confirm that stress conditions, such as ER, oxidative and metabolic stress associated with the mitochondria, are reduced in aggregated blastocysts. These findings may help improve the production of IVP blastocysts with high developmental competence and contribute significantly to biomedical research.

## Materials & Methods

### Chemicals

All chemicals were purchased from Sigma-Aldrich Chemical Co. (St. Louis, MO, USA), unless otherwise noted.

### Oocyte Collection and *in vitro* maturation (IVM)

Porcine ovaries were collected from a nearby local abattoir in 0.9% saline containing 50 µg/mL streptomycin sulfate and 75 µg/mL potassium penicillin G at 38.5 °C within 2 h. Cumulus-oocyte complexes (COCs) were retrieved from follicles (3–7 mm in diameter) using an 18-gauge needle fixed to a disposable 10 mL syringe. COCs were washed three times with Tyrode’s Albumin Lactate Pyruvate-HEPES medium (Funahashi et al., 1994). Next, 50 COCs were cultured in 500 µL IVM medium, which consisted of tissue culture medium 199 containing 10% porcine follicular fluid, 10 ng/mL *β*-mercaptoethanol, 0.57 mM cysteine, 10 ng/mL epidermal growth factor, 10 IU/mL pregnant mare serum gonadotropin (PMSG) and 10 IU/mL human chorionic gonadotropin (hCG) in a 4-well multi-dish (Nunc, Roskilde, Denmark) for 22 h at 38.5 °C in 5% CO_2_ in air. After the first 22 h of IVM, the COCs were transferred in to fresh maturation medium without PMSG and hCG for additional 22 h at 38.5 °C in 5% CO_2_ in air.

### Parthenogenetic activation (PA)

Metaphase II (MII) oocytes were placed in a 1 mm gab wire chamber (CUY 5000P1; Nepa Gene, Chiba, Japan) added with 280 mM mannitol containing 0.5 mM HEPES, 0.1 mM CaCl_2_⋅2H_2_O, 0.1 mM MgSO_4_⋅7H_2_O and 0.01% polyvinyl alcohol (PVA) (Beebe, McIlfatrick & Nottle, 2009). MII oocytes were promptly activated by one direct current pulse of 1.8 kV/cm for 50 µs using an Electro Cell Fusion Generator (LF 101; Nepa gene) and then cultured in porcine zygote medium-3 (PZM-3) supplemented with 2mM 6-dimethylaminopurine and 5 mg/mL cytochalasin B for 4 h at 38.5 °C in 5% CO_2_ in air. Activated oocytes were washed and cultured in PZM-3 at 38.5 °C in 5% CO_2_ in air.

### *In vitro* fertilization (IVF)

IVF was carried out as described previously (Jeong et al., 2017). IVF was performed in modified Tris-buffered medium (mTBM) containing 113.1 mM NaCl, 3 mM KCl, 7.5 mM CaCl_2_⋅2H_2_O, 20 mM Tris (Fisher Scientific, Fair Lawn, NJ, USA), 11 mM glucose, and 5 mM sodium pyruvate, with no antibiotics. MII oocytes were washed three times in mTBM containing 2.5 mM caffeine sodium benzoate and 1 mg/mL bovine serum albumin (BSA), and 10–15 oocytes were placed into 48 µL droplets of IVF medium under mineral oil pre-equilibrated at 38.5 °C in 5% CO_2_ in air. To prepare the spermatozoa using the swim-up method before fertilization, freshly ejaculated semen was washed three times with sperm washing medium (Dulbecco’s phosphate-buffered saline [DPBS; Gibco-BRL, Grand Island, NY, USA] supplemented with 1 mg/mL BSA, 100 µg/mL penicillin G, and 75 µg/mL streptomycin sulfate). After washing, 2 mL of sperm washing medium was gently added to the spermatozoa pellet and incubated for 15 min at 38.5 °C in 5% CO_2_ in air. After incubation, the supernatant was washed with mTBM, and then resuspended with 1 mL of mTBM. Then, 2 µL of diluted spermatozoa was added to 48 µL of mTBM containing 10–15 oocytes to a final concentration of 1.5 × 10^5^ spermatozoa/mL. Oocytes were co-incubated with the spermatozoa for 6 h at 38.5 °C in 5% CO_2_ in air. After 6 h, oocytes were stripped by gentle pipetting and transferred to PZM-3 for culture at 38.5 °C in 5% CO_2_ in air.

### Embryo aggregation method and *in vitro* tracing

Embryo aggregation method was carried out as described previously (Lee et al., 2007). The zona pellucida of 4-cell stage embryos was removed using acidic Tyrode’s solution. Clusters of depressions were generated in the bottom of a culture dish using gentle pressure with a darning needle (BLS, Budapest, Hungary), covered with PZM-3, overlaid with paraffin oil (Junsei, Tokyo, Japan). For embryo aggregation, zona-free embryos were placed into each microwell. Zona-intact (negative control; NC), non-aggregated (1X), two-embryo aggregated (2X), and three-embryo aggregated (3X) 4-cell stage embryos were cultured in parallel in separate drops within the same dish. The aggregates were cultured in PZM-3 for 2 days and then were cultured in PZM-3 supplemented with 10% fetal bovine serum (Gibco-BRL) for 2 days at 38.5 °C in 5% CO_2_ in air (Mun et al., 2017).

To confirm the possibility of embryo aggregation, zona-free 4 cell stage embryos were labeled with fluorescent carbocyanine dye (DiI; red, DiO; green, Takara bio Inc., Shiga, Japan). Zona-free 4 cell stage embryos were washed DPBS containing 4mg/mL BSA and placed in 1% DiI or DiO for 10 min, and then washed with PZM-3 and cultured in aggregation microwells with the darning needle. Fluorescence was observed under a fluorescence microscope (Olympus, Tokyo, Japan).

### Immunocytochemistry

Blastocysts were fixed in 4% paraformaldehyde overnight at 4 °C and washed three times in DPBS with 0.1% PVA (DPBS-PVA). The fixed blastocysts were treated with DPBS containing 0.5% Triton X-100 for 1 h at room temperature (RT), then washed in DPBS-PVA. Next, the blastocysts were incubated in DPBS-PVA supplemented with 1 mg/mL BSA (DPBS-PVA-BSA) at 4 °C overnight and were stored with 10% normal goat serum for 1 h at RT. Primary antibody was used mouse monoclonal anti-Cdx2 (an undiluted solution; Biogenex Laboratories Inc., San Ramon, CA, USA) for overnight at 4 °C. After washed three times with DPBS-PVA-BSA for 10 min and incubated for 1 h at RT with conjugated secondary antibodies, Alexa-Fluor-488-labeled goat anti-mouse IgG (1:200). After washed three times in DPBS-PVA-BSA for 10 min and stained with 2 µg/mL 4′, 6′-diamidino-2-phenylindole (DAPI). DAPI-labeled or Cdx2-positive nuclei were observed using a fluorescence microscope (Olympus).

### Terminal deoxynucleotidyl transferase-mediated dUTP-digoxygenin nick end-labeling (TUNEL) Assay

TUNEL assay was carried out using an *in-situ* cell death detection kit (Roche, Basel, Switzerland). The blastocysts were washed three times in DPBS-PVA and fixed in 4% paraformaldehyde overnight at 4 °C. Fixed blastocysts were permeabilized in DPBS containing 0.5% Triton X-100 at RT for 1 h. Subsequently, blastocysts were washed three times with DPBS-PVA and stained with fluorescein-conjugated dUTP and terminal deoxynucleotidyl transferase for 1 h at 38.5 °C. The blastocysts were washed three times with DPBS-PVA and mounted on clean glass slides with DAPI. DAPI-labeled or TUNEL-positive nuclei were observed under a fluorescence microscope (Olympus).

### Mitochondrial distribution (MitoTracker), mitochondrial membrane potential (JC-1) analysis

The MitoTracker and JC-1 staining were modified carried out as described previously (Yang et al., 2018). Blastocysts were washed with DPBS-PVA and fixed in 4% paraformaldehyde overnight at 4 °C. Fixed blastocysts were washed three times and stained with 0.8 µM MitoTracker green (Invitrogen, CA, USA) and JC-1 (100:1) (Cayman Chemical, MI, USA) for 30 min at 38.5 °C. JC-1 staining expresses two types of fluorescence. The aggregated form (J-aggregate; favoured at high membrane potential) of mitochondria indicated red fluorescence, whereas and the monomers form (J-monomer; favoured at low membrane potential) indicated green fluorescence. Therefore, it is possible to use the ratio of red to green fluorescence to determine mitochondrial membrane potential. After the blastocysts were washed three times in DPBS-PVA for 10 min. each, the DNA was stained with 2 µg/mL DAPI. DAPI-labeled nuclei or MitoTracker or JC-1 were observed using a fluorescence microscope (Olympus). The quantification of fluorescence levels was measured using ImageJ software (version 1.47; National Institute of Health, Bethesda, MD, USA) after normalization through subtraction of the background intensity from each embryo size.

### Measurement of intracellular ROS

Measurement of ROS levels in embryos were carried out as described previously (Mun et al., 2017). Intracellular ROS levels were detected by 5 µM 5-(and-6)-chloromethyl-2′,7′-dichlorodihydro-fluorescein diacetate, acetyl ester (CM-H2DCFDA; Invitrogen) and the blastocysts were washed three times with DPBS-PVA. Fluorescence was observed under a fluorescence microscope (DMI 4000B; Leica, Wetzlar, Germany) with ultraviolet filters (460 nm). The quantification of fluorescence levels was measured using ImageJ software after normalization through subtraction of the background intensity from each embryo size.

### Quantitative real-time polymerase chain reaction (qRT-PCR)

Poly(A) mRNAs were extracted using the Dynabeads mRNA Direct kit (Invitrogen) from 20 blastocysts according to the manufacturer’s protocol. The resulting poly(A) mRNAs were reverse transcribed in 20 µL reactions containing 5 × RT buffer (containing 25 mM Mg^2+^), oligo(dT)_20_, 10 mM mixture of dNTPs and 10 U of the RNase inhibitor ReverTra Ace (Toyobo, Osaka, Japan). The following PCR conditions were used: 95 °C for 30 s, 60 °C for 30 s and 72 °C for 30 s, followed by extension at 72 °C for 5 min. qRT-PCR were performed with SYBR premix Ex Taq (Takara Bio Inc.) using Mx3000P QPCR system (Agilent, Santa Clara, CA, USA). The sample delta Ct (^*S*Δ*CT*^) value was calculated from the difference between the Ct values of GAPDH the target genes. The relative gene expression levels between the samples and the controls were determined using the formula 2^−(*S*Δ*CT*−*C*Δ*CT*)^. The primers used in the present study are listed in Table S1.

### Analysis of mitochondrial DNA copy number

Analysis of mitochondrial DNA copy number was carried out as described previously (Itami et al., 2018). The mitochondrial DNA copy number from 10 blastocysts represents the average mitochondrial DNA copy number of an individual donor blastocyst. The mitochondrial DNA copy number was determined by DNA extraction and qRT-PCR using a Mx3000P QPCR system (Agilent) with the primer set (5′-CGAGAAAGCACTTTCCAAGG-3′ and 5′-CTAATTCGGGTGTTGGTGCT-3′). The primers were designed using Primer3Plus and the sequence data for porcine mitochondria (Accession number AF304202) to amplify a 151-base pair. Melting curve was analyzed to verify the specificity of the PCR products, followed by electrophoresis to determine the product size. As an external standard, the PCR product of the corresponding gene was cloned into a vector using the Zero Blunt TOPO PCR cloning kit (Invitrogen). The product was sequenced for confirmation before use.

### Statistical analysis

The blastocyst formation rate (blastocyst formation number per cultured embryos) and proportion of blastocysts by diameter (blastocyst number of each indicated size per total blastocysts) were recorded as the percentage. The cell numbers within the blastocysts were counted by DAPI- labeled or Cdx2- positive nuclei (Cdx2 expressing cell is TE, and opposite is ICM). Apoptosis (apoptotic cell number per total cell number by TUNEL assay) was recorded as the percentage. The number of independent replicates (Re) of each experiment is shown in the figure legends. Data are expressed as the mean ± standard error of the mean (SEM). Data were compared using analysis of variance (ANOVA), followed by Duncan’s multiple range test using SigmaStat Software (Systat Software Inc., San Jose, CA, USA). *P*-values less than 0.05 were considered to indicate statistical significance.

## Results

### Production of aggregated blastocysts using porcine 4-cell stage embryos

**Figure 1:**
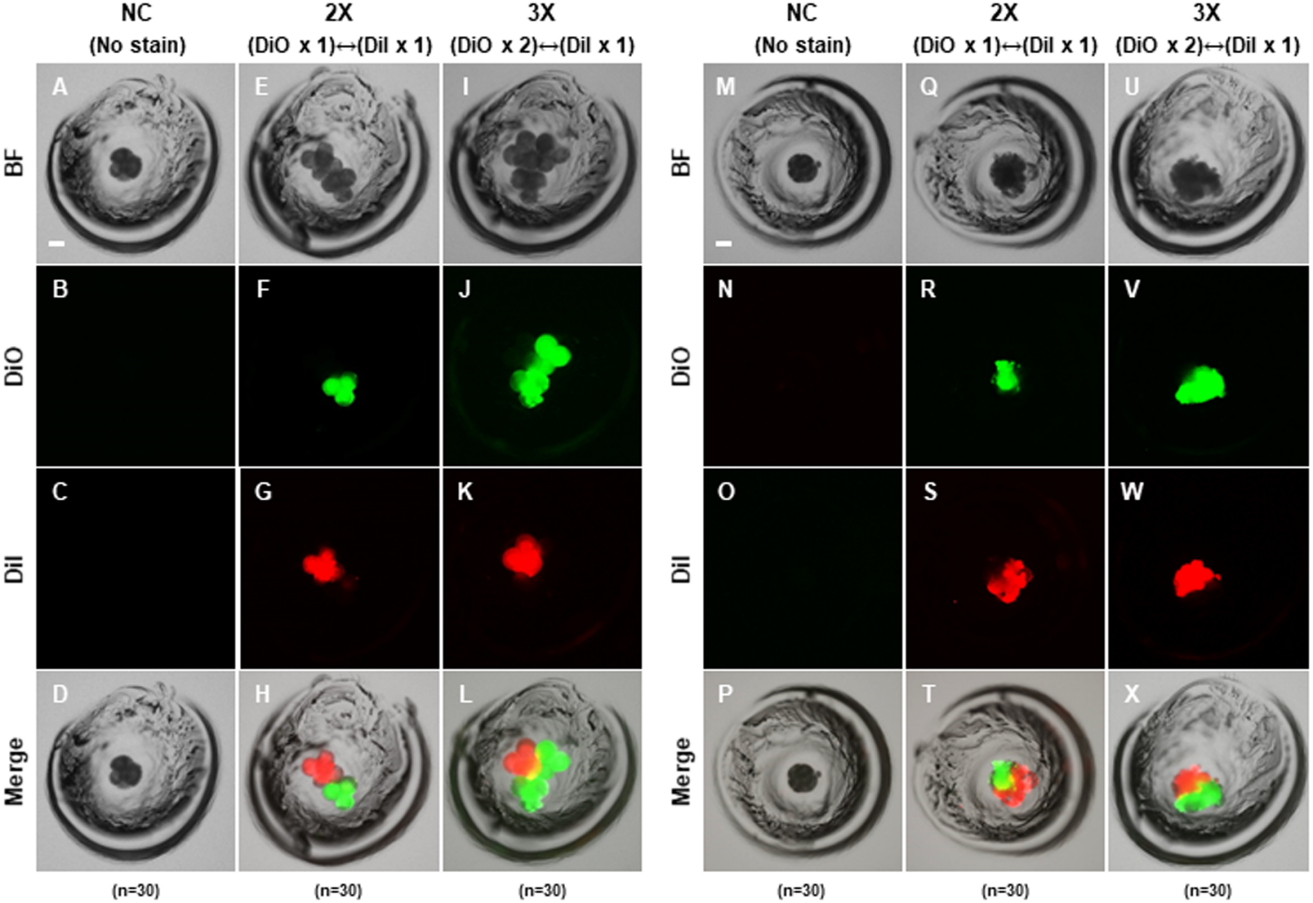
Aggregation of porcine PA embryos at 4-cell stage. Morphological and fluorescent images showing the aggregation between the used embryos after (A–L) 24 h and (M–X) 48 h. Green and red fluorescence indicate used embryos labeled with fluorescent carbocyanine dye DiI or DiO, respectively. Bar = 50 um. For all panels, *n* indicates number of embryos examined. Re = 3. NC; negative control (zona-intact), 2X; two zona-free embryos, 3X; three zona-free embryos.

**Figure 2:**
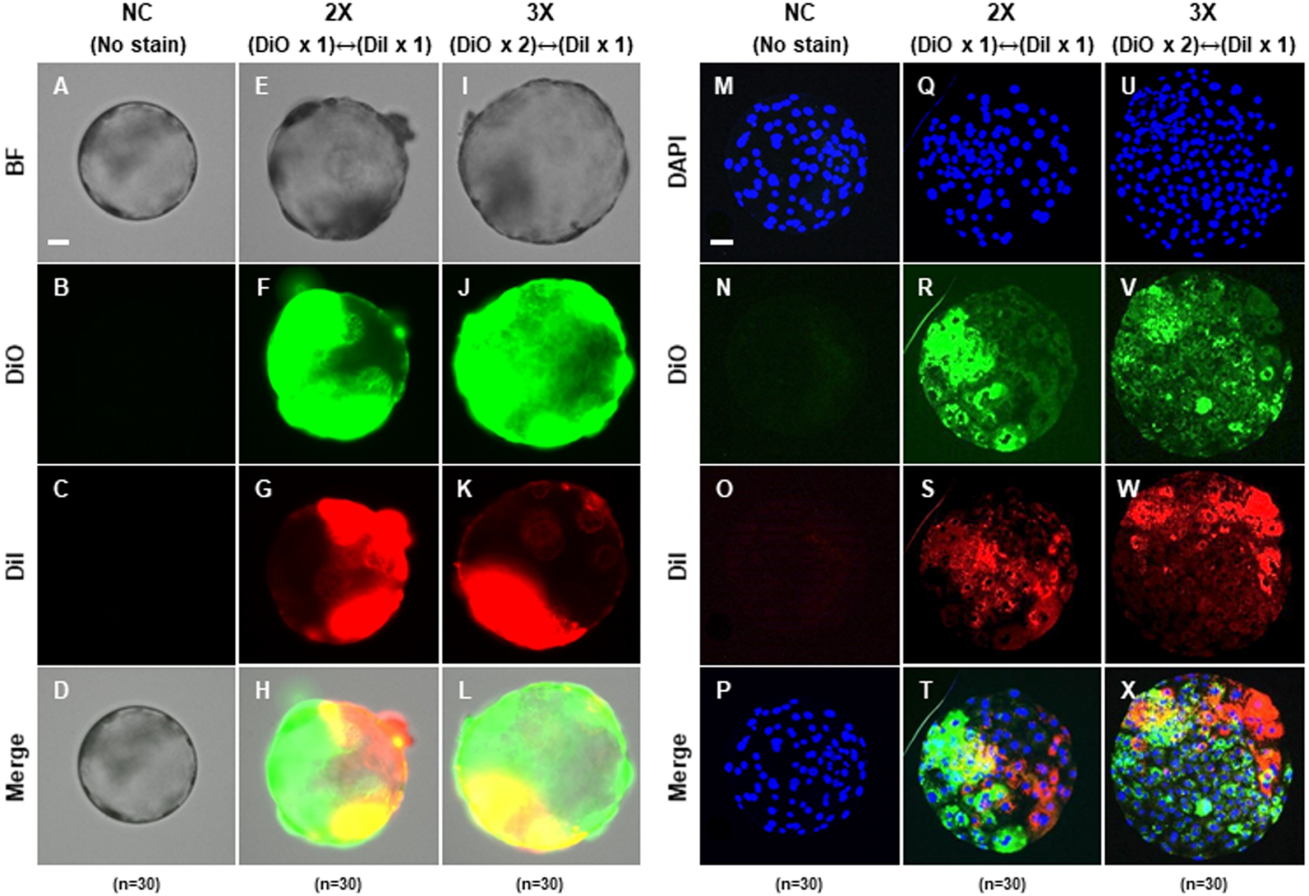
Blastocysts derived from two or three 4-cell stage embryos at 72 h after aggregation. (A–L) Representative photographs of blastocysts derived from 2X and 3X embryos labeled with fluorescent carbocyanine dye DiI or DiO, respectively. Bar = 50 um. (M–X) Aggregation pattern of used embryo for blastocysts formation. Nuclear staining of blastocysts aggregated with 2X and 3X embryo labeled with fluorescent carbocyanine dye DiI or DiO, respectively. Bar = 50 um. For all panels, *n* indicates number of embryos examined. Re = 3. NC; negative control (zona-intact), 2X; two zona-free embryos, 3X; three zona-free embryos.

To confirm that the PA embryos could aggregate, we cultured zona-intact (negative control; NC), two-embryo aggregated (2X) and three-embryo aggregated (3X) using only 4-cell stage embryos, respectively. The zona pellucida of 4-cell stage embryos was removed by treatment with acidic Tyrode’s solution, and then differentially incubated with DiI (red) or DiO (green) for membrane staining. Embryo aggregation was observed in the morula and blastocyst stages derived from 2X and 3X embryos (Figs. 1M–1X and Figs. 2A–2L). Fluorescence of DiI or DiO was observed during the 4-cell, morula and blastocyst stages in 2X and 3X, but not in the negative control (Figs. 1A–1X and Figs. 2A–2L). Fluorescence of DiI and DiO was observed separately in the 4-cell and morula stages (Figs. 1A–1X), while DiI and DiO overlapping fluorescence was observed in the blastocyst stage (Figs. 2A–2L). To confirm the precise DiI or DiO patterns, aggregation-derived blastocysts were fixed and observed; however, no consistent pattern by two fluorescent markers was confirmed, indicating that two or three embryos were randomly aggregated (Figs. 2M–2X). These results indicated that blastocysts could be obtained via aggregation using two or three PA embryos.

### Effect of embryo aggregation on developmental competence in porcine IVP embryos

**Figure 3:**
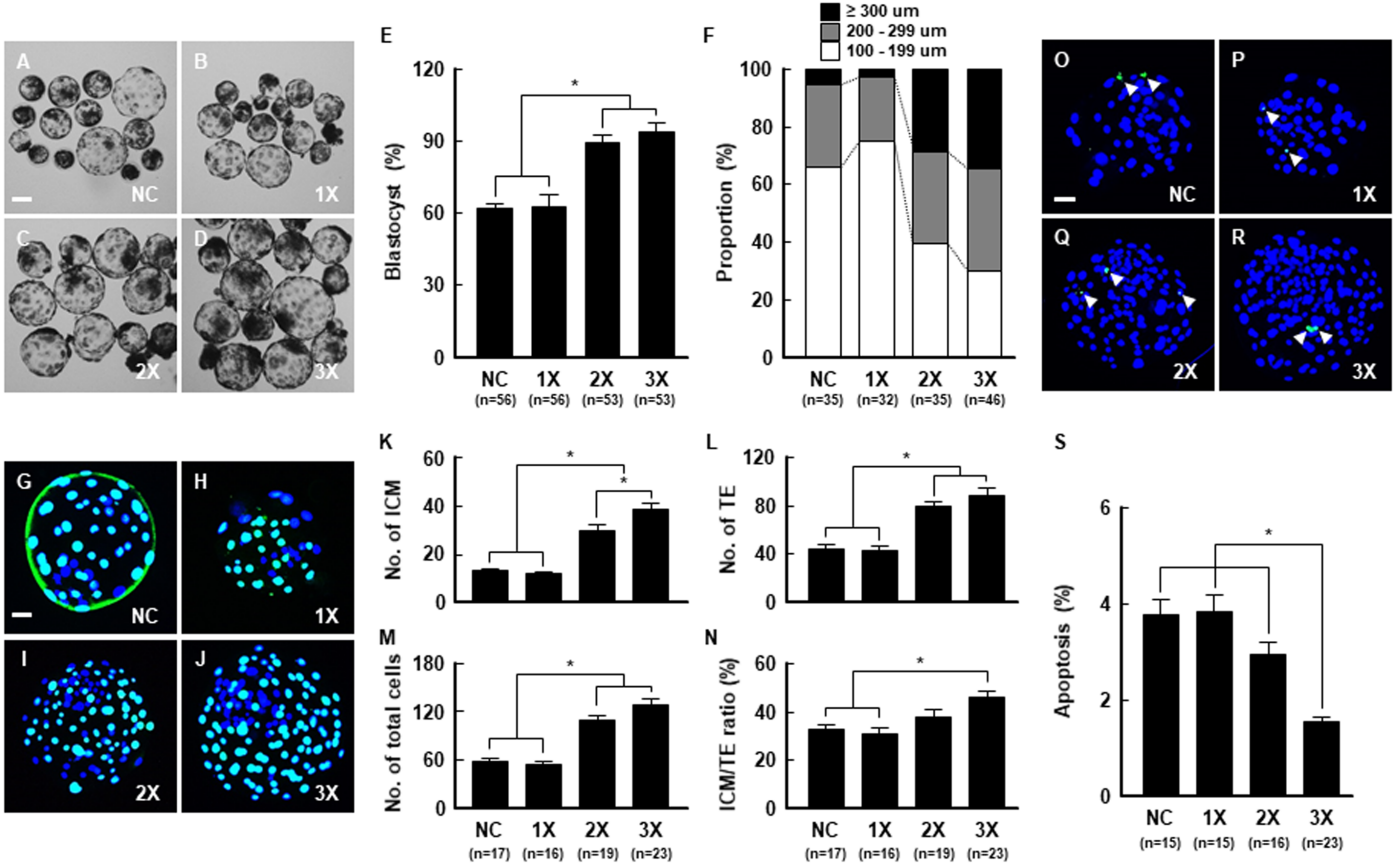
Effect of embryo aggregation on developmental competence of porcine PA embryos. (A–D) Representative photographs of blastocysts developed from the indicated embryo number for aggregation. Bar = 100 um. (E) Blastocyst formation rates in the indicated groups. (*P < 0.05). (F) Proportion by blastocysts diameter in the indicated groups. (G–J) Immunocytochemistry of Cdx2/DAPI using blastocysts developed in the indicated groups. Merged images between DAPI (blue) and Cdx2 (green) signals are shown. Bar = 50 um. (K–N) Quantification of the total, ICM, TE cell numbers, and ICM/TE ratios in the indicated groups. (*P < 0.05). (O–R) TUNEL assay using blastocysts developed in the indicated groups. Merged images (light green) between DAPI (blue) and TUNEL (green, white arrow) signals are shown. Bar = 50 um. (S) Quantification of proportion of apoptotic cells in the indicated groups. (*P < 0.05). For all panels, *n* indicates number of embryos examined. Re = 3. NC; negative control (zona-intact), 1X; one zona-free embryo, 2X; two zona-free embryos, 3X; three zona-free embryos.

To investigate the effect of aggregation on the developmental competence of porcine embryos, PA embryos (NC, 1X, 2X and 3X) were tested for aggregation in microwells. Compared to the NC, 1X embryos showed no detrimental effect on blastocyst formation upon removal of the zona pellucida. Meanwhile, 2X and 3X aggregated embryos showed significant increases in blastocyst formation rate (Figs. 3A–3E; Table S2) and blastocyst diameter (Fig. 3F; Table S3) compared to the NC and 1X groups. Next, we assessed the quality of the aggregated embryos using Cdx2 staining and the TUNEL assay. Blastocysts derived from 2X and 3X embryos showed significantly increased total ICM and TE cell numbers, and the ratio of ICM cells against TE cells was significantly higher in the 3X group (Figs. 3G–3N; Table S4). The 3X group also exhibited a markedly decreased apoptotic cell rate (Figs. 3O–3S; Table S5). To define whether embryo aggregation enhanced developmental competence in IVF embryos, we conducted aggregation using 4-cell stage embryos derived from IVF, which were cultured to the blastocyst stage. Consistent with the developmental results of the PA embryos, 3X aggregated embryos showed significantly improved blastocyst formation rates and blastocyst diameters compared to 1X embryos (Figs. S1A–S1D; Tables S6 and S7). Moreover, the total cell number and ICM/TE ratio increased markedly in the 3X group (Figs. S1E–S1J; Table S8), along with cellular survival (S1K–S1M; Table S9). These findings confirmed that aggregation of 4-cell stage embryos derived from both IVF and PA could improve the developmental competence in porcine early embryogenesis. Therefore, we conducted subsequent experiments using PA embryos for embryo aggregation to prevent variations in embryo aggregation due to sperm factors associated with IVF, such as polyspermy.

### Regulation of intracellular ROS levels and mitochondrial function by embryo aggregation

**Figure 4:**
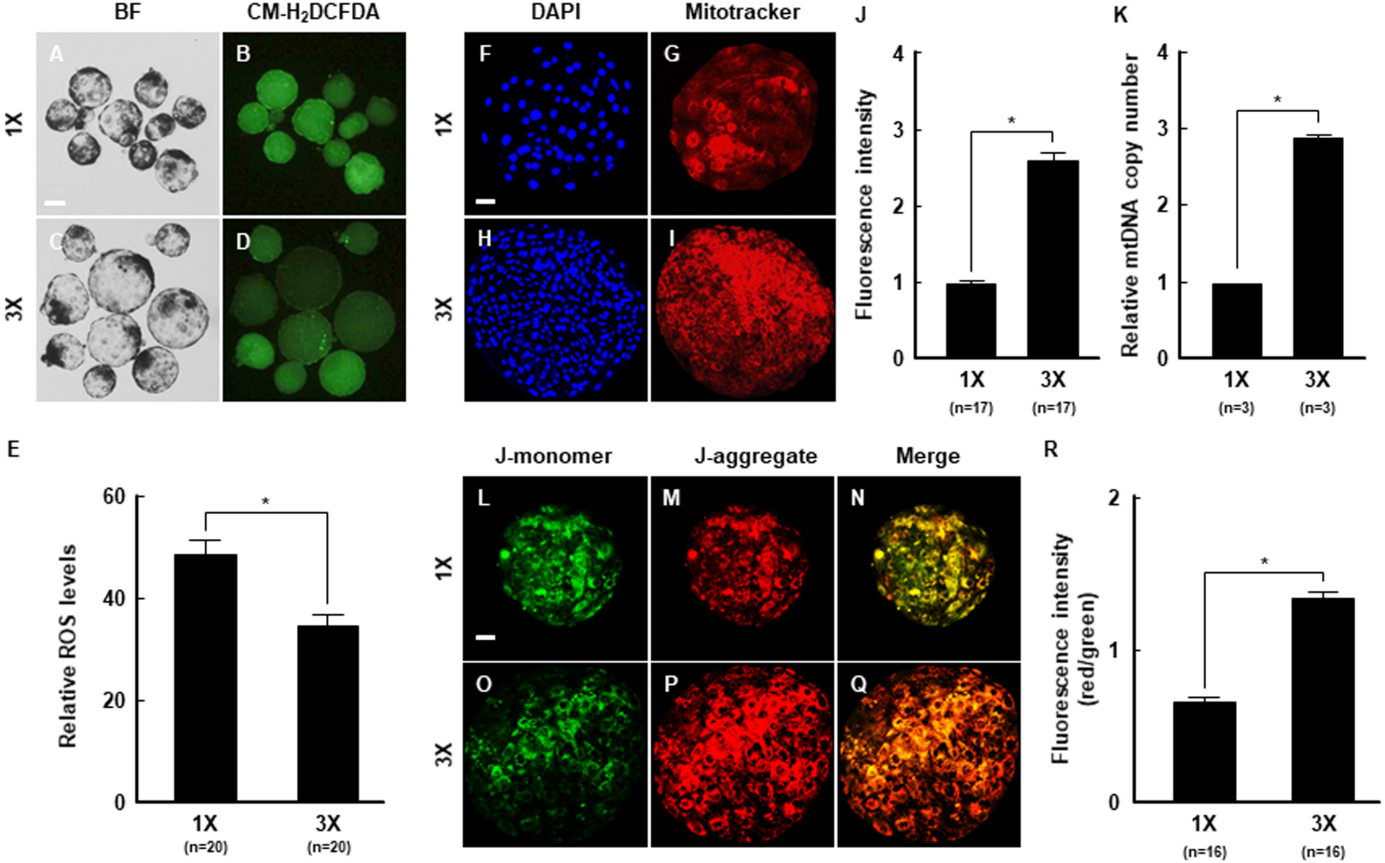
The beneficial effects of embryo aggregation on mitochondrial function. (A–D) Fluorescence images of blastocysts treated with CM-H_2_DCFDA for measurement of intracellular ROS level in the indicated groups. Bar = 100 um. (E) Quantification of the fluorescence intensity in the indicated groups. (*P < 0.05). (F–I) Fluorescent images of blastocysts showing MitoTracker (red) and DAPI (blue) staining in the indicated groups. Bar = 50 um. (J) Quantification of the fluorescence intensity in the indicated groups. (*P < 0.05). (K) Quantification of the mitochondrial copy number using real-time PCR in the indicated groups. (*P < 0.05). (L–Q) Expression of J-monomer (green) and J-aggregate (red) were stained by JC-1 staining at the blastocysts in the indicated groups. Bar = 50 um. (R) Quantification of the fluorescence intensity (red/green) in the indicated groups. (*P < 0.05). For all panels, *n* indicates number of embryos examined. Re = 3. 1X; one zona-free embryo, 3X; three zona-free embryos.

To investigate changes in intracellular ROS and mitochondrial function that affected developmental competence by embryo aggregation, intracellular ROS levels were measured in blastocysts derived from 1X and 3X embryos using CM-H_2_DCFDA staining and fluorescence microscopy. ROS levels were markedly lower in the 3X than 1X group (Figs. 4A–4E). We also assessed the distribution and membrane potential of mitochondria in blastocysts to confirm that the increase in ROS was associated with mitochondrial dysfunction, as described in previous studies (Niu et al., 2017). Mitochondria were evenly distributed in the blastocysts (Figs. 4F–4I), and MitoTracker staining confirmed that fluorescence intensity was significantly higher in the 3X than 1X group (Fig. 4J). These results are consistent with the significant increase in mitochondria DNA copy number (Fig. 4K). Moreover, the J-aggregate (high membrane potential)/J-monomer (low membrane potential) ratio, which indicates mitochondrial membrane potential, was higher in the 3X than 1X group (Figs. 4F–4R). This further confirmed that embryo aggregation prominently improved intracellular ROS levels and elevation of the mitochondrial function in porcine embryos.

### Regulation of transcription levels related to stress conditions and embryonic development by embryo aggregation

**Figure 5:**
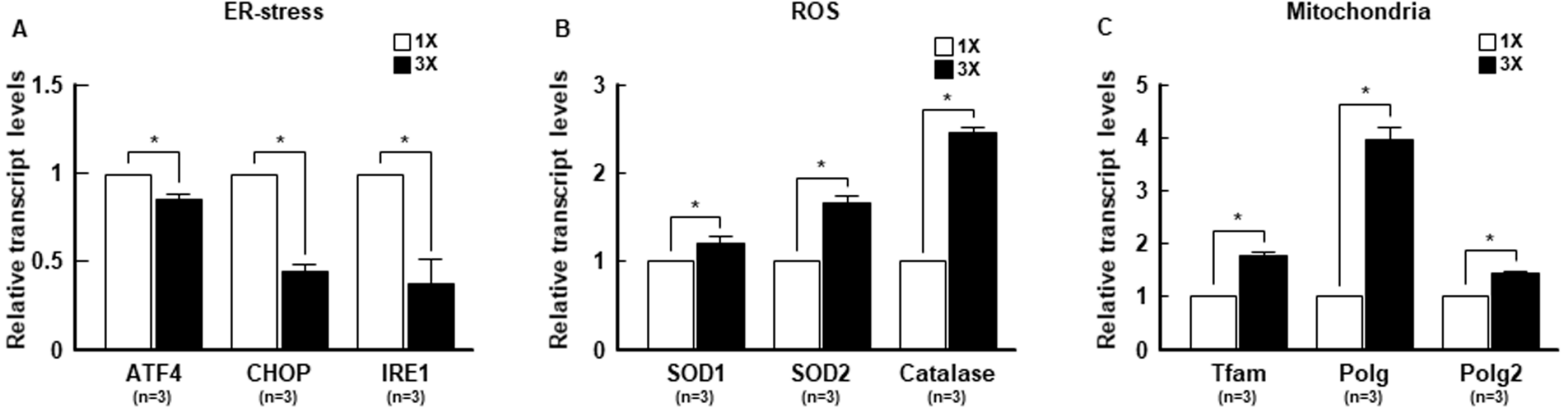
The effect of embryo aggregation on transcript levels of stress-related genes. Real-time PCR results for (A) ER-stress related genes (*ATF4*, *CHOP*, *IRE1*), (B) ROS related genes (*SOD1*, *SOD2*, *Catalase*) and (C) mitochondria related genes (*TFAM*, *POLG*, *POLG2*) at blastocysts in the indicated groups. All genes were normalized with the *GAPDH* gene. (*P < 0.05). For all panels, *n* indicates number of embryos examined. Re = 3. 1X; one zona-free embryo, 3X; three zona-free embryos.

**Figure 6:**
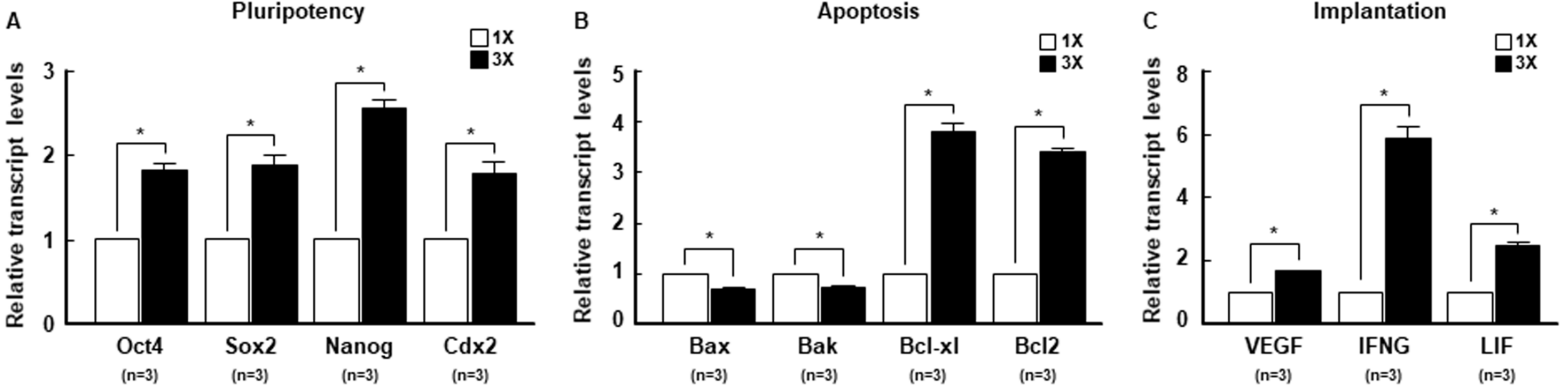
The effect of embryo aggregation on transcription levels of embryonic development-related genes. Real-time PCR results for (A) pluripotent related genes (*Oct4*, *Sox2*, *Nanog*, *Cdx2*), (B) apoptosis related genes (*Bax*, *Bak*, *Bcl-xl*, *Bcl2*) and (C) implantation related genes (*VEGF*, *IFNG*, *LIF*) at blastocysts in the indicated groups. All genes were normalized with the *GAPDH* gene. (*P < 0.05). For all panels, *n* indicates number of embryos examined. Re = 3. 1X; one zona-free embryo, 3X; three zona-free embryos.

To investigate the molecular mechanism underlying the increase in developmental competence by embryo aggregation, we examined the transcription levels of key modulators of stress conditions, such as ER-stress (Gupta et al., 2010), ROS (Mun et al., 2017; Yoon et al., 2014) and mitochondrial function (Spikings, Alderson & St John, 2007), using real-time PCR. Embryo aggregation resulted in a significant downregulation of ER stress-related genes, such as *ATF4*, *CHOP* and *IRE1* (Fig. 5A), and significant upregulation of antioxidant-related genes, such as *SOD1*, *SOD2*, and *catalase* (Fig. 5B). In addition, the expression of mitochondrial function-related genes was significantly upregulated in the 3X group compared to the 1X group (Fig. 5C). Next, we investigated whether embryo aggregation modulated mRNA expression of embryonic development-related genes, such as pluripotency (Wu & Scholer, 2014), apoptosis (Gupta et al., 2010), and implantation (Guzeloglu-Kayisli, Kayisli & Taylor, 2009). Expression of pluripotency and anti-apoptosis related genes were significantly upregulated and pro-apoptosis-related genes were downregulated in the 3X group (Figs. 6A and 6B). Moreover, the mRNA expression of implantation related genes was considerably increased by aggregation (Fig. 6C). Taken together, these results demonstrate that embryo aggregation significantly increased embryonic development by reducing stress conditions in porcine early embryogenesis.

## Discussion

IVP embryos with high developmental competence are necessary to generate transgenic pigs. Therefore, much attention has focused on improving the quality of IVP embryos. Despite these efforts, the developmental competence of IVP embryos remains low, and consequently, the pregnancy rates and yields of live-born offspring is relatively low. To overcome these problems, we demonstrated that developmental competence increased in porcine IVP embryos by embryo aggregation via reducing the stress conditions and improving mitochondrial function.

Embryo aggregation is widely used in the production of transgenic and chimeric animals in various mammals, including mice (Yamaguchi et al., 2017), bovine (Simmet et al., 2015) and monkeys (Tachibana et al., 2012), to improve the production efficiency by compensating for the developmental deficiency of IVP embryos. In particular, pigs have been reported to respond positively to embryo aggregation, with increases in blastocyst formation rate, total cell number, ICM/TE ratio, and cellular survival (Lee et al., 2007; Terashita et al., 2011). Also, the blastocysts derived from embryo aggregation promoted the efficiency of pluripotent embryonic stem cells (Lee et al., 2015). Recently, chimeric pigs were produced by aggregation using cloned 4-cell stage embryos (Huang et al., 2016). We showed similar results in which the embryo aggregation method was adequate for 4-cell stage embryos, significantly enhancing key parameters of developmental competence, such as blastocyst formation rate, total cell number, ICM/TE ratio, cellular survival and expression of pluripotency, apoptosis, and implantation related genes. Thus, we propose that embryo aggregation helps to increase the quality of pre-implantation embryos, leading to successful post-implantation development in pigs.

Several studies have reported that ROS accumulation decreases the developmental competence of porcine embryos by inducing DNA damage and pro-apoptotic gene expression (Bain, Madan & Betts, 2011; Takahashi, 2012). Furthermore, increased ROS levels cause mitochondrial dysfunction, which reduces mitochondrial membrane potential (Chen, Chomyn & Chan, 2005). It has been shown that mitochondrial DNA is associated with fertilization outcome and early porcine embryogenesis, and mitochondrial DNA deficiency has a negative effect on normal oocyte maturation, with impaired oocytes being restored by mitochondrial supplementation (Cagnone et al., 2016). In addition, mitochondrial membrane potential is required not only for pre-implantation embryo development but also for post-implantation outcomes (Wakefield, Lane & Mitchell, 2011). In this study, ROS levels were significantly lower in 3X than 1X blastocysts. These results are consistent with the increased mitochondrial DNA copy numbers and increased mitochondrial membrane potential in aggregated blastocysts. In particular, our results demonstrated that the expression of *SOD1*, *SOD2* and *catalase*, genes related to ROS, were substantially increased in 3X blastocysts, and the mRNA quantity of mitochondrial membrane potential-related genes was significantly increased compared to 1X blastocysts. These results strongly suggest that embryo aggregation can enhance porcine embryo development through the reduction of intracellular ROS levels and the promotion of mitochondrial function.

Unwanted stress conditions, especially ER stress, generally act as developmental barriers in IVP embryo development. Studies have shown that embryo development is blocked by treatment with tunicamycin (an ER stress activator) but restored by treatment with tauroursodeoxycholic acid (TUDCA; an ER stress inhibitor) (Kim et al., 2012; Zhang et al., 2012). In this study, we observed that 3X blastocysts decreased transcription of ER stress-related genes, including *ATF4*, *CHOP*, and *IRE1*, compared to 1X blastocysts. Interestingly, TUDCA treatment increased the ICM/TE ratio in porcine IVP blastocysts (Kim et al., 2012; Zhang et al., 2012). Previous studies showed that aggregation-derived blastocysts increased the ICM/TE ratio and *oct4* transcripts; however, the underlying reasons for this remain unknown (Buemo et al., 2016; Siriboon et al., 2014; Terashita et al., 2011). Similarly, we showed that 3X blastocysts significantly improved not only the ICM/TE ratio, but also *oct4* transcripts. Therefore, embryo aggregation may affect ER stress reduction, which induces the improvement of developmental competence in porcine early embryogenesis.

Interactions between the ER and mitochondria have been reported to be related to the regulation of the Ca^2+^ signaling pathway, energy metabolism, and cellular survival (Berridge, 2002). Changes in cellular Ca^2+^ occur as a result of ER stress, thereby increasing ROS production and decreasing the mitochondrial membrane potential. It was recently shown that mitochondrial permeability transition (MPT) involved the ER stress-induced apoptosis signaling pathway, including the Bcl-2 family via the release of cytochrome C (Wu et al., 2012). The Bcl-2 protein family are known to localize in both the ER and the mitochondria, because they contain shared Bcl-2 homology (BH) domains. Anti-apoptotic proteins such as Bcl-2 and Bcl-xl prevent ER stress-induced mitochondrial damage by transducing the BH4 domain (Gupta et al., 2010). Meanwhile, Bax and Bak, which are pro-apoptotic proteins that share a BH3 domain, induce MPT, resulting in ER stress-induced mitochondrial damage and cell death (Gupta et al., 2010). In this study, we demonstrated embryo aggregation as an effective method to reduce the expression of Bax and Bak and increase Bcl-2 and Bcl, as confirmed by the reduction in apoptosis related to ER stress, ROS, and mitochondrial function.

## Conclusions

Embryo aggregation significantly reduced stress conditions, such as ER, oxidative, and metabolic stress, indicating an improvement in developmental competence during porcine early embryogenesis. Furthermore, improved developmental competence by embryo aggregation enhanced the expression of pluripotency, anti-apoptosis, and implantation-related genes, which improved post-implantation development potential. Our findings suggest that embryo aggregation is a valuable tool for producing IVP embryos with high developmental competence, thereby aiding the production of chimeric and transgenic pigs for biomedical research.

##  Supplemental Information

10.7717/peerj.8143/supp-1 Figure S1 Effect of embryo aggregation on developmental competence of porcine IVF embryos (A, B) Representative photographs of blastocysts developed from the indicated group for aggregation. Bar = 100 um. (C) Blastocyst formation rates in the indicated groups. (*P ¡ 0.05). (D) Proportion by blastocysts diameter in the indicated groups. (E, F) Immunocytochemistry of Cdx2/DAPI using blastocysts developed in the indicated groups. Merged images between DAPI (blue) and Cdx2 (green) signals are shown. Bar = 50 um. (G-J) Quantification of the total, ICM, TE cell numbers, and ICM/TE ratios in the indicated groups. (*P ¡ 0.05). (K, L) TUNEL assay using blastocysts developed in the indicated groups. Merged images (light green) between DAPI (blue) and TUNEL (green, white arrow) signals are shown. Bar = 50 um. (M) Quantification of proportion of apoptotic cells in the indicated groups. (*P ¡ 0.05). For all panels, *n* indicates number of embryos examined. Re = 3. 1X; one zona-free embryo, 3X; three zona-free embryos

10.7717/peerj.8143/supp-2 Table S1 Primer sequences for RT-PCR

10.7717/peerj.8143/supp-3 Table S2 Effect of zona-free embryo number on aggregation in porcine PA embryos Data are the mean ± SEM, and values with different superscript letter within a column differ significantly (*p* < 0.05).

10.7717/peerj.8143/supp-4 Table S3 Effect of zona-free embryo number on blastocyst diameter in aggregated-porcine PA blastocysts Data are the mean ± SEM, and values with different superscript letter within a column differ significantly (*p* ¡ 0.05).

10.7717/peerj.8143/supp-5 Table S4 Effect of zona-free embryo number on ICM/TE proportion in aggregated-porcine PA blastocysts Data are the mean ± SEM, and values with different superscript letter within a column differ significantly (*p* ¡ 0.05).

10.7717/peerj.8143/supp-6 Table S5 Effect of zona-free embryo number on cellular survival in aggregated-porcine PA blastocysts Data are the mean ± SEM, and values with different superscript letter within a column differ significantly (*p* ¡ 0.05).

10.7717/peerj.8143/supp-7 Table S6 Effect of zona-free embryo number on aggregation in porcine IVF embryos Data are the mean ± SEM, and values with different superscript letter within a column differ significantly (*p* ¡ 0.05).

10.7717/peerj.8143/supp-8 Table S7 Effect of zona-free embryo number on blastocyst diameter in aggregated-porcine IVF blastocysts Data are the mean ± SEM, and values with different superscript letter within a column differ significantly (*p* ¡ 0.05).

10.7717/peerj.8143/supp-9 Table S8 Effect of zona-free embryo number on ICM/TE proportion in aggregated-porcine IVF blastocysts Data are the mean ± SEM, and values with different superscript letter within a column differ significantly (*p* ¡ 0.05).

10.7717/peerj.8143/supp-10 Table S9 Effect of zona-free embryo number on cellular survival in aggregated-porcine IVF blastocysts Data are the mean ± SEM, and values with different superscript letter within a column differ significantly (*p* ¡ 0.05).

10.7717/peerj.8143/supp-11 Data S1 Raw data for the statistic analysis Raw data applied for data analyses and preparation for Figure 3B, 3C, 3E and 3G; Figure 4B, 4D, 4E and 4G; Figure 5A, 5B and 5C; Figure 6A, 6B and 6C; Supplementary figure 1B, 1C, 1E and 1G.

## Additional Information and Declarations

### Data Availability

The following information was supplied regarding data availability: The raw measurements are available in the Supplemental Files.
