# Supplementary figures and images for: Embryo aggregation regulates in vitro stress conditions to promote developmental competence in pigs
Source: PeerJ. 2019 Dec 13;7:e8143. doi: 10.7717/peerj.8143 (PMC6913270; doi:10.7717/peerj.8143)

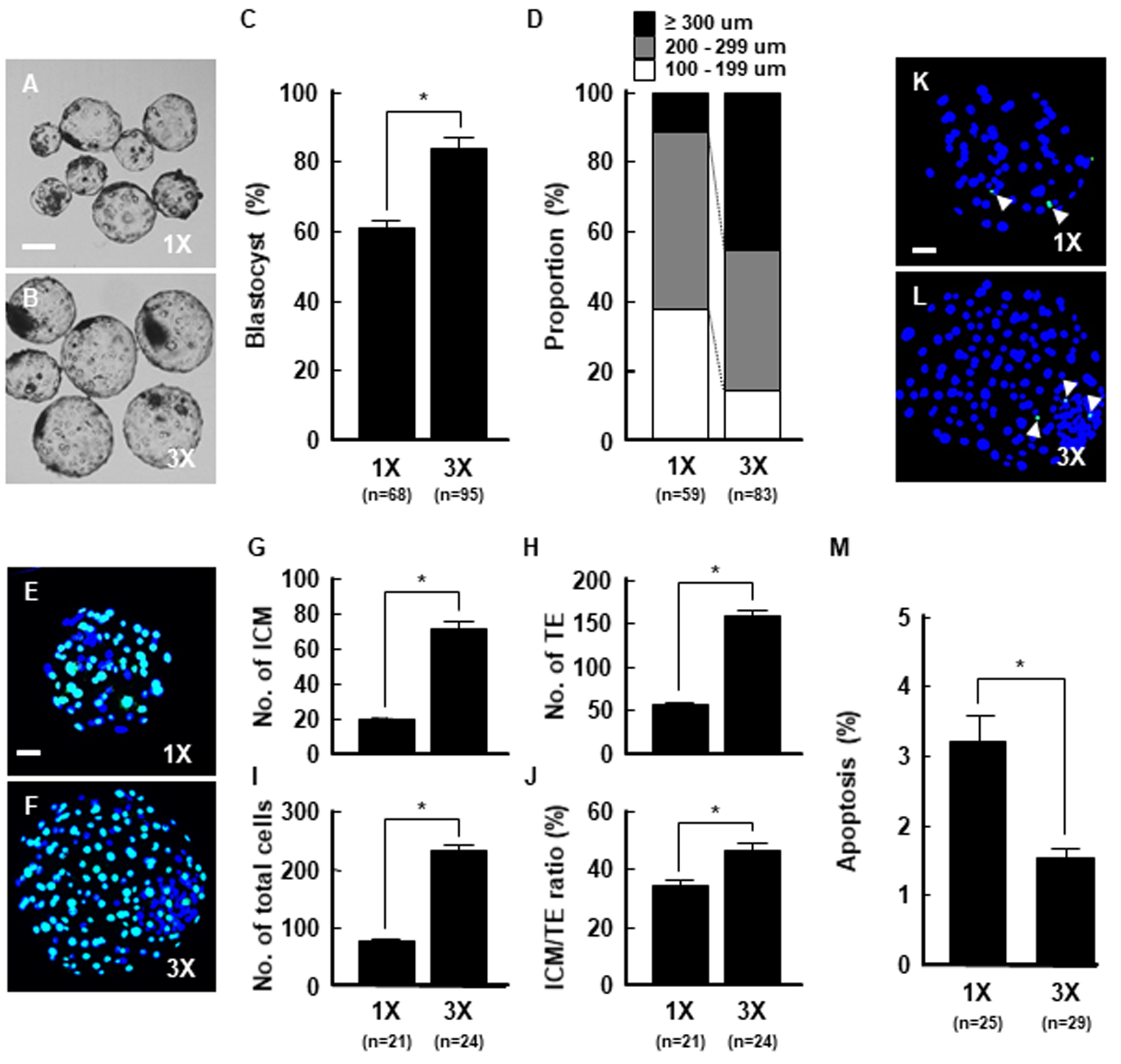

Supplement: Figure S1 — (A, B) Representative photographs of blastocysts developed from the indicated group for aggregation. Bar = 100 um. (C) Blastocyst formation rates in the indicated groups. (*P ¡ 0.05). (D) Proportion by blastocysts diameter in the indicated groups. (E, F) Immunocytochemistry of Cdx2/DAPI using blastocysts developed in the indicated groups. Merged images between DAPI (blue) and Cdx2 (green) signals are shown. Bar = 50 um. (G-J) Quantification of the total, ICM, TE cell numbers, and ICM/TE ratios in the indicated groups. (*P ¡ 0.05). (K, L) TUNEL assay using blastocysts developed in the indicated groups. Merged images (light green) between DAPI (blue) and TUNEL (green, white arrow) signals are shown. Bar = 50 um. (M) Quantification of proportion of apoptotic cells in the indicated groups. (*P ¡ 0.05). For all panels, n indicates number of embryos examined. Re = 3. 1X; one zona-free embryo, 3X; three zona-free embryos [file peerj-07-8143-s001.png]
