# Supplementary material for: Embryo aggregation regulates in vitro stress conditions to promote developmental competence in pigs
Source: PeerJ. 2019 Dec 13;7:e8143. doi: 10.7717/peerj.8143 (PMC6913270; doi:10.7717/peerj.8143)
Supplement: Table S1 [file peerj-07-8143-s002.docx]

Supplementary table S1 Primer sequences for RT-PCR

| Gene | Primer sequences | GenBank  accession no. | Product  size (bp) |
| --- | --- | --- | --- |
| *ATF4* | F: 5’- CAA CAG CAA GGA GGA TGC TTT -3’ | NM_001123078.1 | 112 |
|  | R: 5’- TGG CAT GGT TTC CAG GTC AT -3’ |  |  |
| *CHOP* | F: 5’- AGG AGG TGC TGT CCT CAG AT -3’ | NM_001144845.1 | 149 |
|  | R: 5’- GGA GGT GTG TGT GAC CTC TG -3’ |  |  |
| *IRE1* | F: 5’- CTC TAT GCG TCG CCC TCA AT -3’ | XM_005668695.1 | 149 |
|  | R: 5’- AAC TTC AGG TCT GTG CTG GG -3’ |  |  |
| *SOD1* | F: 5’- GGT GGG CCA AAG GAT CAA GA -3’ | NM_001190422.1 | 80 |
|  | R: 5’- TAC ACA GTG GCC ACA CCA TC -3’ |  |  |
| *SOD2* | F: 5’- GGT GGA GGC CAC ATC AAT CA -3’ | NM_214127.2 | 219 |
|  | R: 5’- AAC AAG GGC AAT CTG CAA G -3’ |  |  |
| *Catalase* | F: 5’- TGT ACC CGC TAT TCT GGG GA -3’ | NM_214301.2 | 119 |
|  | R: 5’- TCA CAC AGG CGT TTC CTC TC -3’ |  |  |
| *TFAM* | F: 5’- TCA GTG CTT TGT CTA CGG GT -3’ | NM_001130211.1 | 110 |
|  | R: 5’- CAG TCG ACT TCC ACA AAC CG -3’ |  |  |
| *POLG* | F: 5’- GCT TCC CTC TGC ACT ACT CA -3’ | XM_001927064.5 | 160 |
|  | R: 5’- TGC TCA AGA CAG TGC TTC CT -3’ |  |  |
| *POLG2* | F: 5’- GTC ATC GAC TCC TGT GGT GG -3’ | XM_021066763.1 | 98 |
|  | R: 5’- AAG TTT GTT TCC CTT CCG GC -3’ |  |  |
| *Oct4* | F: 5’- AGT GAG AGG CAA CCT GGA GA -3’ | NM_001113060.1 | 151 |
|  | R: 5’- ACT GCT TGA TCG TTT GCC CT -3’ |  |  |
| *Sox2* | F: 5’- AGC CCA GAC CGA GTT AAG CG -3’ | NM_001123197.1 | 85 |
|  | R: 5’- TGG GGT TCT CTT GGG CCA TC -3’ |  |  |
| *Nanog* | F: 5’- GGT TCC AGA ACC AGC GAA TGA -3’ | XM_021092390.1 | 93 |
|  | R: 5’- CTG TAC TGG CTG AGC CCT GA -3’ |  |  |
| *Cdx2* | F: 5’- GGC AGC CAA GTG AAA ACC AG -3’ | NM_001278769.1 | 119 |
|  | R: 5’- GCC TTT CTC CGA ATG GTG AT -3’ |  |  |
| *Bax* | F: 5’- CGA TCT CGA AGG AAG TCC AG -3’ | XM_003127290.5 | 251 |
|  | R: 5’- AAG CGC ATT GGA GAT GAA CT -3’ |  |  |
| *Bak* | F: 5’- CTA GAA CCT AGC AGC ACC AT -3’ | XM_001928147.3 | 151 |
|  | R: 5’- CGA TCT TGG TGA AGT ACT C -3’ |  |  |
| *Bcl-xl* | F: 5’- AGG GCA TTC AGT GAC CTG AC -3’ | NM_214285.1 | 242 |
|  | R: 5’- TGG ATC CAA GGC TCT AGG TG -3’ |  |  |
| *Bcl2* | F: 5’- GGA GGG GAC ACT CTT CTT CC -3’ | XM_021099593.1 | 189 |
|  | R: 5’- CTG GGC ACA ATT GGT AGC TT -3’ |  |  |
| *VEGF* | F: 5’- ACA TCT TCA AGC CGT CCT GT -3’ | NM_001130211.1 | 110 |
|  | R: 5’- TAT GTG CTG GCC TTG GTG AG -3’ |  |  |
| *IFNG* | F: 5’- GGA GCA TGG ATG TGA TCA AG -3’ | XM_001927064.5 | 160 |
|  | R: 5’- GAG TTC ACT GAT GGC TTT GC -3’ |  |  |
| *LIF* | F: 5’- TCA CTG CGC ATC AGT TCT TC -3’ | XM_005670785.3 | 190 |
|  | R: 5’- TGT GCG ACG TCA TAT GGT CT -3’ |  |  |
| *GAPDH* | F: 5’- CCC TGA GAC ACG ATG GTG AA -3’ | NM_001206359.1 | 147 |
|  | R: 5’- GGA GGT CAA TGA AGG GGT CA -3’ |  |  |
