# Supplementary material for: Embryo aggregation regulates in vitro stress conditions to promote developmental competence in pigs
Source: PeerJ. 2019 Dec 13;7:e8143. doi: 10.7717/peerj.8143 (PMC6913270; doi:10.7717/peerj.8143)
Supplement: Table S2 — Data are the mean ± SEM, and values with different superscript letter within a column differ significantly (p < 0.05). [file peerj-07-8143-s003.docx]

Supplementary table S2. Effect of zona-free embryo number on aggregation in porcine PA embryos

| Groups | No. of embryos examined  (aggregated embryos) | Aggregated (%) | Blastocyst (%) |
| --- | --- | --- | --- |
| NC | 56 | N.A | 35 (62.3±1.5)^a^ |
| 1X | 56 | N.A | 36 (63.3±5.1)^a^ |
| 2X | 106 (53) | 46 (86.3±3.2) | 41 (89.6±3.2)^b^ |
| 3X | 159 (53) | 52 (97.8±2.2) | 49 (93.6±4.2)^b^ |

Data are the mean ± SEM, and values with different superscript letter within a column differ significantly (*p* < 0.05).
