# Supplementary material for: Embryo aggregation regulates in vitro stress conditions to promote developmental competence in pigs
Source: PeerJ. 2019 Dec 13;7:e8143. doi: 10.7717/peerj.8143 (PMC6913270; doi:10.7717/peerj.8143)
Supplement: Table S3 — Data are the mean ± SEM, and values with different superscript letter within a column differ significantly (p ¡ 0.05). [file peerj-07-8143-s004.docx]

Supplementary table S3. Effect of zona-free embryo number on blastocyst diameter in aggregated-porcine PA blastocysts

| Groups | No. of embryos examined | Blastocyst diameter (%) | | |
| --- | --- | --- | --- | --- |
|  |  | 100 um - 199 um | 200 um – 299 um | ≥ 300 um |
| NC | 35 | 66.1±2.5^a^ | 28.6±1.8^a,b^ | 5.3±2.7^a^ |
| 1X | 32 | 75.1±2.4^b^ | 21.8±2.8^a^ | 3.0±3.0^a^ |
| 2X | 35 | 40.1±0.9^c^ | 31.4±1.0^b^ | 28.6±1.8^b^ |
| 3X | 46 | 30.4±1.5^d^ | 34.8±0.7^b^ | 34.8±0.7^b^ |

Data are the mean ± SEM, and values with different superscript letter within a column differ significantly (*p* < 0.05).
