# Supplementary material for: Embryo aggregation regulates in vitro stress conditions to promote developmental competence in pigs
Source: PeerJ. 2019 Dec 13;7:e8143. doi: 10.7717/peerj.8143 (PMC6913270; doi:10.7717/peerj.8143)
Supplement: Table S4 — Data are the mean ± SEM, and values with different superscript letter within a column differ significantly (p ¡ 0.05). [file peerj-07-8143-s005.docx]

Supplementary table S4. Effect of zona-free embryo number on ICM/TE proportion in aggregated-porcine PA blastocysts

| Groups | No. of blastocysts examined | No. of nuclei | | | ICM/TE (%) |
| --- | --- | --- | --- | --- | --- |
|  |  | ICM | TE | Total |  |
| NC | 17 | 13.2±1.0^a^ | 43.5±3.9^a^ | 56.8±4.6^a^ | 32.1±2.0^a^ |
| 1X | 16 | 12.0±0.9^a^ | 42.6±4.0^a^ | 54.6±4.2^a^ | 30.7±2.8^a^ |
| 2X | 19 | 30.0±2.9^b^ | 79.3±4.8^b^ | 109.3±7.0^b^ | 38.0±2.9^a,b^ |
| 3X | 23 | 38.8±2.3^c^ | 89.1±6.1^b^ | 127.9±7.7^b^ | 46.1±2.8^b^ |

Data are the mean ± SEM, and values with different superscript letter within a column differ significantly (*p* < 0.05).
