# Supplementary material for: Embryo aggregation regulates in vitro stress conditions to promote developmental competence in pigs
Source: PeerJ. 2019 Dec 13;7:e8143. doi: 10.7717/peerj.8143 (PMC6913270; doi:10.7717/peerj.8143)
Supplement: Table S5 — Data are the mean ± SEM, and values with different superscript letter within a column differ significantly (p ¡ 0.05). [file peerj-07-8143-s006.docx]

Supplementary table S5. Effect of zona-free embryo number on cellular survival in aggregated-porcine PA blastocysts

| Groups | No. of blastocysts examined | No. of total cells | No. of TUNEL-positive cells | Apoptosis (%) |
| --- | --- | --- | --- | --- |
| NC | 15 | 45.6±1.8^a^ | 1.7±0.2 | 3.8±0.3^a^ |
| 1X | 15 | 47.5±2.4^a^ | 1.8±0.2 | 3.8±0.4^a^ |
| 2X | 16 | 103.4±8.5^b^ | 2.9±0.3 | 3.0±0.3^a,b^ |
| 3X | 23 | 149.9±9.2^c^ | 2.3±0.2 | 1.6±0.1^b^ |

Data are the mean ± SEM, and values with different superscript letter within a column differ significantly (*p* < 0.05).
