# Supplementary material for: Embryo aggregation regulates in vitro stress conditions to promote developmental competence in pigs
Source: PeerJ. 2019 Dec 13;7:e8143. doi: 10.7717/peerj.8143 (PMC6913270; doi:10.7717/peerj.8143)
Supplement: Table S6 — Data are the mean ± SEM, and values with different superscript letter within a column differ significantly (p ¡ 0.05). [file peerj-07-8143-s007.docx]

Supplementary table S6. Effect of zona-free embryo number on aggregation in porcine IVF embryos

| Groups | No. of embryos examined  (aggregated embryos) | Aggregated (%) | Blastocyst (%) |
| --- | --- | --- | --- |
| 1X | 112 | N.A | 68 (61.1±1.7)^a^ |
| 3X | 336 (112) | 111 (99.4±0.6) | 95 (84.3±2.6)^b^ |

Data are the mean ± SEM, and values with different superscript letter within a column differ significantly (*p* < 0.05).
