# Supplementary material for: Embryo aggregation regulates in vitro stress conditions to promote developmental competence in pigs
Source: PeerJ. 2019 Dec 13;7:e8143. doi: 10.7717/peerj.8143 (PMC6913270; doi:10.7717/peerj.8143)
Supplement: Table S7 — Data are the mean ± SEM, and values with different superscript letter within a column differ significantly (p ¡ 0.05). [file peerj-07-8143-s008.docx]

Supplementary table S7. Effect of zona-free embryo number on blastocyst diameter in aggregated-porcine IVF blastocysts

| Groups | No. of embryos examined | Blastocyst diameter (%) | | |
| --- | --- | --- | --- | --- |
|  |  | 100 um - 199 um | 200 um – 299 um | ≥ 300 um |
| 1X | 59 | 37.9±4.1^a^ | 50.3±3.5^a^ | 11.7±0.6^a^ |
| 3X | 83 | 14.7±2.8^b^ | 39.9±1.8^b^ | 45.5±2.9^b^ |

Data are the mean ± SEM, and values with different superscript letter within a column differ significantly (*p* < 0.05).
