# Supplementary material for: Embryo aggregation regulates in vitro stress conditions to promote developmental competence in pigs
Source: PeerJ. 2019 Dec 13;7:e8143. doi: 10.7717/peerj.8143 (PMC6913270; doi:10.7717/peerj.8143)
Supplement: Table S8 — Data are the mean ± SEM, and values with different superscript letter within a column differ significantly (p ¡ 0.05). [file peerj-07-8143-s009.docx]

Supplementary table S8. Effect of zona-free embryo number on ICM/TE proportion in aggregated-porcine IVF blastocysts

| Groups | No. of blastocysts examined | No. of nuclei | | | ICM/TE (%) |
| --- | --- | --- | --- | --- | --- |
|  |  | ICM | TE | Total |  |
| 1X | 21 | 19.6±1.2^a^ | 57.0±3.0^a^ | 76.6±4.0^a^ | 34.7±1.5^a^ |
| 3X | 24 | 72.4±3.8^b^ | 159.1±7.4^b^ | 231.5±9.6^b^ | 46.6±2.4^b^ |

Data are the mean ± SEM, and values with different superscript letter within a column differ significantly (*p* < 0.05).
