# Supplementary material for: Embryo aggregation regulates in vitro stress conditions to promote developmental competence in pigs
Source: PeerJ. 2019 Dec 13;7:e8143. doi: 10.7717/peerj.8143 (PMC6913270; doi:10.7717/peerj.8143)
Supplement: Table S9 — Data are the mean ± SEM, and values with different superscript letter within a column differ significantly (p ¡ 0.05). [file peerj-07-8143-s010.docx]

Supplementary table S9. Effect of zona-free embryo number on cellular survival in aggregated-porcine IVF blastocysts

| Groups | No. of blastocysts examined | No. of total cells | No. of TUNEL-positive cells | Apoptosis (%) |
| --- | --- | --- | --- | --- |
| 1X | 25 | 76.2±3.9^a^ | 2.2±0.3^a^ | 3.2±0.4^a^ |
| 3X | 29 | 219.0±9.6^b^ | 3.2±0.2^b^ | 1.5±0.1^b^ |

Data are the mean ± SEM, and values with different superscript letter within a column differ significantly (*p* < 0.05).
